# A CNN-Transformer Hybrid Framework for Multi-Label Predator–Prey Detection in Agricultural Fields

**DOI:** 10.3390/s25154719

**Published:** 2025-07-31

**Authors:** Yifan Lyu, Feiyu Lu, Xuaner Wang, Yakui Wang, Zihuan Wang, Yawen Zhu, Zhewei Wang, Min Dong

**Affiliations:** 1 China Agricultural University, Beijing 100083, China; 2 University of International Business and Economics, Beijing 100029, China; 3 Peking University, Beijing 100871, China; 4 College of Plant Protection, China Agricultural University, Beijing 100083, China

**Keywords:** intelligent pest control, predator–prey detection, multi-label recognition, agricultural computer vision, co-attention mechanism

## Abstract

Accurate identification of predator–pest relationships is essential for implementing effective and sustainable biological control in agriculture. However, existing image-based methods struggle to recognize insect co-occurrence under complex field conditions, limiting their ecological applicability. To address this challenge, we propose a hybrid deep learning framework that integrates convolutional neural networks (CNNs) and Transformer architectures for multi-label recognition of predator–pest combinations. The model leverages a novel co-occurrence attention mechanism to capture semantic relationships between insect categories and employs a pairwise label matching loss to enhance ecological pairing accuracy. Evaluated on a field-constructed dataset of 5,037 images across eight categories, the model achieved an F1-score of 86.5%, mAP50 of 85.1%, and demonstrated strong generalization to unseen predator–pest pairs with an average F1-score of 79.6%. These results outperform several strong baselines, including ResNet-50, YOLOv8, and Vision Transformer. This work contributes a robust, interpretable approach for multi-object ecological detection and offers practical potential for deployment in smart farming systems, UAV-based monitoring, and precision pest management.

## 1. Introduction

With the rising demand for sustainable agricultural practices, effective pest control is facing growing pressure to reduce chemical inputs while maintaining crop yields. Manual pest monitoring remains the dominant approach in many regions, yet it is labor-intensive, time-consuming, and prone to subjectivity. Inadequate or delayed identification of pest outbreaks not only increases economic losses—estimated to affect 20–40% of global crop yields—but also leads to the excessive use of pesticides, which harms beneficial insect populations and threatens long-term ecological balance. In this context, biological control strategies, particularly those leveraging natural enemies such as predatory insects, have emerged as promising alternatives due to their environmental safety and species specificity [1,2]. These natural control methods have been widely applied across fruits, vegetables, and grains, offering a viable path toward reducing pesticide use and enhancing ecological resilience [3]. However, the practical deployment of biological control still faces key limitations, including low identification efficiency, imprecise matching of predators and pests, and weak system integration. These issues highlight the urgent need for intelligent automation tools to support scalable and accurate ecological monitoring. Within integrated pest management frameworks, decisions about natural enemy species selection, release timing, and spatial targeting depend heavily on the reliable recognition of predator–pest interactions [4,5]. Nonetheless, image data collected in real field conditions pose substantial challenges: cluttered backgrounds, inconsistent lighting, and wide scale variation among insect targets degrade detection quality [6,7]. Compounding this difficulty is the high inter-species similarity between pests and their natural enemies in color, texture, and morphology, which often renders traditional single-object classifiers unreliable [8]. Moreover, since pests and their predators frequently co-occur in complex spatial or temporal patterns, recognizing individual insects in isolation is insufficient to capture their ecological relationships—limiting the ability of conventional models to inform precise pest management decisions.

Several studies have begun to explore the application of deep learning methods in insect image recognition. Among these, convolutional neural networks (CNNs) have been widely used in scenarios such as field pest recognition and insect classification due to their powerful local perception capabilities [9,10,11]. For instance, Venkateswara S M et al. proposed a deep learning-based pest monitoring method that identifies 82 pest species using the IP102 dataset. Data imbalance was addressed via autoencoder-generated augmentation, while RGB encoding and object detection enabled pest localization and segmentation. The method achieved an IoU of 80% and a classification accuracy of 84.95%, surpassing existing models [12]. Yulita I N et al. developed a GoogleNet-based detection model integrated into a mobile app for assisting Indonesian farmers, attaining a classification accuracy of 93.78%, the best in their study [13]. Coulibaly S et al. introduced a CNN-based method for detecting 102 pest species using over 75,000 images from the IP102 dataset. Explainable visualizations of color and shape features facilitated pest localization and user interaction. Mutual information scoring guided network optimization, reducing parameters by 58.90% and enhancing interpretability and efficiency [14]. In recent years, with the widespread adoption of Transformers in the visual domain, visual Transformer architectures have been increasingly applied to pest detection in complex backgrounds due to their superior global modeling capabilities [15]. Peng et al. introduced a hybrid method combining Transformers and convolutional modules, achieving classification accuracies of 74.897% and 75.583% on the IP102 dataset at 224×224 and 480×480 resolutions, and 99.472% and 97.935% on the D0 and Li’s datasets, respectively. This method effectively leverages spatial modeling to outperform mainstream approaches in complex scenes [16]. Nevertheless, most existing approaches remain focused on the recognition and classification of individual objects, lacking the capacity to model the latent semantic relationships among multiple insect targets within an image. This deficiency is particularly evident in the task of “natural enemy–pest” pairing, where challenges such as incomplete recognition, inaccurate matching, and absence of ecological logic are widespread [17,18]. Additionally, the scarcity of publicly available multi-label datasets featuring “natural enemy–pest” pairings further constrains the deployment and advancement of deep learning methods in biological control scenarios.

To address the aforementioned challenges, a multi-label recognition framework is proposed, integrating CNN and Transformer architectures for the simultaneous detection and semantic pairing of common natural enemies and target pests in field-captured images. This method incorporates attention mechanisms to explicitly model the co-occurrence relationships among various targets within an image, constructing a “natural enemy–pest” pairing graph that combines local perception and global semantic modeling. The framework employs a lightweight convolutional module to extract fine-grained insect features and utilizes multi-head self-attention to achieve coupled representation of natural enemy and pest features. The output structure includes a multi-label classification branch and a relational modeling head to predict interaction matching strength between targets. During training, a label co-occurrence matrix and ecological prior knowledge are introduced to enhance the model’s capability in recognizing rare pairings and improving generalization. At the data level, the first multi-label “natural enemy–pest” pairing image dataset based on real field scenarios was constructed, covering economically significant crops such as tomato, pepper, and maize. The dataset includes more than 15 natural enemy classes and 20 common pest classes, with image collection conducted through on-site sampling and collaboration with local farmers, significantly enhancing the realism and representativeness of the data. This method demonstrates robust cross-species generalization and resilience in visually complex environments, providing effective support for agricultural intelligent recognition systems. The main contributions of this study are as follows:

1. **Forward-looking problem formulation**: The deficiency in co-occurrence relationship modeling within current insect recognition methods is systematically identified, and for the first time, the integrated modeling of multi-label recognition and semantic pairing for “natural enemy–pest” relationships is explicitly proposed.
2. **Architecture integrating perception and relational modeling**: A CNN-Transformer hybrid structure is designed to combine local feature extraction with global relationship modeling, significantly enhancing object recognition and pairing performance in complex imagery.
3. **Construction of an ecological multi-label dataset**: The first publicly available multi-label “natural enemy–pest” pairing dataset based on natural field scenes is developed, featuring high ecological fidelity and strong task relevance, providing a solid foundation for future research.
4. **Application-oriented methodology**: The proposed method exhibits strong precision, adaptability, and interpretability, offering technical support for intelligent biological control systems and contributing to the implementation of precision agriculture and green pest management.

## 2. Related Work

### 2.1. Agricultural Pest and Disease Image Recognition Research Status

In recent years, deep learning has achieved significant progress in the field of agricultural pest and disease image recognition, particularly in the detection and classification of single pest species. CNNs, due to their advantage in local feature extraction, have become the mainstream models widely applied in this domain. For instance, Rahman et al. applied VGG16 and InceptionV3 with transfer learning on real rice field images, achieving robust performance. A lightweight two-stage CNN was also designed for mobile use, reaching 93.3% accuracy with a 99% reduction in size [19]. Additionally, Guo et al. developed a YOLOv3-based method for automatic rice pest detection, attaining a mAP of 91.93% across five pest types, and up to 97.40% for Diostrombus politus Uhler. This method outperformed Faster-RCNN and SSD in both accuracy and speed under complex field conditions [20]. Despite promising achievements in pest and disease recognition, most existing studies remain focused on the detection of individual pest species, often overlooking the complex interactions among species within agroecosystems. For example, limited research attention has been given to the identification of natural enemy insects, which play a critical role in ecological pest control and are essential for ecological agriculture management. Chiranjeevi S et al. proposed InsectNet, a global–local model combining large-scale insect datasets with expert annotations, achieving over 96% accuracy in identifying ecologically relevant insects such as pollinators, parasitoids, predators, and pests under complex backgrounds [8]. To date, systematic studies on effective joint monitoring of pests and natural enemies in real agricultural environments are lacking.

### 2.2. Multi-Object Detection and Co-Occurrence Modeling Methods

Multi-object detection and co-occurrence modeling have become active research areas in the field of object detection, particularly relevant in complex agricultural scenes where the coordinated identification of pests and natural enemy insects is increasingly essential [21]. Traditional object detection models, including YOLO [22], ResNet [23], and Transformer-based architectures [24], have made notable progress, especially in multi-label classification, image-entity co-occurrence recognition, and cross-species identification tasks.

YOLOv8 (You Only Look Once), the latest version in the YOLO series, introduces more efficient CNN designs and multi-scale feature fusion strategies, demonstrating excellent performance in real-time object detection. Yaseen et al. introduced YOLOv9, which outperformed YOLOv8 in mAP and inference speed on the COCO dataset. By integrating GELAN, PGI, depthwise convolutions, and the lightweight C3Ghost structure, the method reduced computational complexity while improving accuracy [25]. However, although YOLOv8 demonstrates strong detection capabilities for agricultural pests, it exhibits limitations in sensitivity to subtle interspecies differences. In scenarios involving the co-occurrence of multiple pest types, it may lead to false positives or missed detections [26]. ResNet has shown strong performance in multi-label classification tasks, and many studies have proposed solutions for multi-object and cross-species recognition based on it. Hu et al. reviewed deep learning-based image recognition methods in weed detection and highlighted the robust performance of deep neural networks, particularly ResNet, in multi-object recognition. These findings provide a methodological foundation for future research in cross-species, multi-label weed detection in farmlands [27]. Despite its achievements in label prediction, ResNet-based approaches often lack sufficient modeling capability for spatial relationships among targets. In complex backgrounds, object overlap and interference may reduce recognition accuracy. Transformer models, known for their strong global modeling capability, have been increasingly applied in object detection and cross-species recognition tasks. Fang et al. introduced Pest-ConFormer, a hybrid CNN-Transformer model incorporating a multi-scale weakly supervised feature selection mechanism. On the large-scale, multi-class IP102 crop pest recognition dataset, an accuracy of 77.81% was achieved, nearly 2 percentage points higher than existing methods. By integrating convolutional and Transformer structures, the method realizes bidirectional modeling of global and local features, significantly enhancing generalization and classification performance in complex natural environments. These results provide strong evidence of the potential of Transformer models in cross-species recognition tasks [28]. However, Transformers face challenges when handling high-resolution images, including high computational cost and reduced real-time performance. In complex agricultural scenes characterized by multi-species co-occurrence and noisy backgrounds, recognition accuracy may deteriorate [29].

Although existing multi-object detection and co-occurrence modeling methods have achieved certain successes in agriculture, they still face significant limitations in handling complex co-occurrence scenarios, fine-grained recognition, and interspecies interactions.

### 2.3. Biological Control of Pests and Intelligent Matching Research

Biological control has gained increasing attention in agricultural pest management as an environmentally friendly strategy. In particular, research on the interactions between natural enemies and pests has become a key focus within the biological control domain [30]. Common natural enemy–pest pairs, such as Trichogramma vs. corn borers and Orius laevigatus vs. thrips, effectively suppress pest populations through parasitism or predation, significantly reducing pesticide usage and promoting ecological balance.

Trichogramma, a widely used parasitic wasp, primarily controls corn borers by parasitizing their eggs. With the growing demand for precision agricultural management, the accurate monitoring of natural enemy–pest interactions has become a crucial topic. El-Faki et al. proposed two image processing methods (ALGO1 and ALGO2) for detecting Trichogramma bourarachae parasitism on Cadra cautella eggs, achieving classification accuracies of 92% and 96% on a 40-image validation set [31]. Despite its advantages in detection efficiency, the method exhibits limited capability in identifying complex interaction scenarios between natural enemies and pests and does not account for population dynamics or multiple influencing factors. The biological control mechanism between Orius laevigatus and Thrips tabaci has also attracted research interest. Orius laevigatus is a significant predatory natural enemy with strong inhibitory effects on thrips [32]. Wang D et al. proposed an improved YOLOv4-based method for detecting small pests, achieving a detection mAP of 92.7% on a yellow trap image dataset for thrips. This result surpassed YOLOv3 and original YOLOv4 by 8.2% and 3.4%, respectively, demonstrating high robustness [33]. However, current methods still lack precision in modeling the spatiotemporal relationships between natural enemies and pests. In dynamic biological control applications, alternating presence and behavioral changes of both parties are often difficult to capture accurately [34].

Although progress has been made in studying natural enemy–pest pairings in biological control, most existing research remains at the stage of individual species monitoring and recognition. Image-driven intelligent pairing methods are still lacking. Existing technologies have not fully leveraged the capabilities of deep learning and computer vision to enable dynamic identification and precise pairing of natural enemies and pests. Therefore, developing image-driven intelligent matching systems will facilitate a deeper understanding and management of biological control relationships, advancing sustainable pest management in precision agriculture.

## 3. Materials and Method

### 3.1. Data Collection

**Figure 1:**
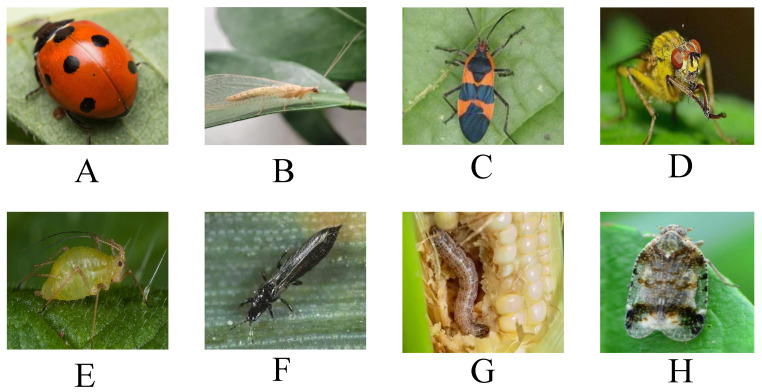
Figure (**A**–**H**) present representative insect samples collected in the dataset. Parts (**A**–**D**) correspond to key natural enemies: *Coccinella septempunctata*, *Chrysoperla sinica*, *Orius sauteri*, and *Trichogramma chilonis*. Parts (**E**–**H**) depict their primary target pests, including *Aphis gossypii*, *Thrips tabaci*, *Ostrinia furnacalis*, and *Nilaparvata lugens*.

The field image acquisition process for this study was conducted from April 2023 to September 2024 across four representative agricultural sites: the experimental fields of China Agricultural University in Changping (Beijing), the maize demonstration zone in Bayannur (Inner Mongolia), the vegetable production base in Shuangfeng (Hunan), and the rice pest monitoring area in Xinghua (Jiangsu), as well as supplementary sources from the Internet. The dataset focuses on four common natural enemy insects—*Coccinella septempunctata*, *Chrysoperla sinica*, *Orius sauteri*, and *Trichogramma chilonis*—and their respective target pests, including *Aphis gossypii*, *Thrips tabaci*, *Ostrinia furnacalis*, and *Nilaparvata lugens* (see Table 1 and Figure 1). To capture diverse ecological interactions, two complementary imaging platforms were utilized: a Sony A7M4 camera for close-range, high-resolution image capture, and a DJI Phantom 4 RTK drone for medium- and long-range monitoring of group-level insect distributions. These platforms inherently introduced variations in lighting conditions, camera angles, and sensor characteristics. To ensure consistency and quality across sources, several measures were implemented: (1) camera parameters such as exposure, ISO, and white balance were pre-calibrated across locations; (2) macro lighting and multi-angle manual tracking were applied during close-range shooting to enhance clarity and reduce motion blur; and (3) overexposed, underexposed, or blurry samples were filtered during post-capture screening. Additionally, EXIF metadata—such as timestamp, illumination level, ambient temperature, and humidity—was retained to support environment-aware analysis and potential model conditioning in future work. To further mitigate cross-platform discrepancies during training, image normalization and augmentation strategies were applied, including brightness jittering, random cropping, and geometric transformations. These steps enhanced the model’s ability to generalize across acquisition conditions. The dataset was divided into three categories: (1) single-object images of natural enemies captured under controlled or close-field conditions, useful for extracting clean semantic features; (2) co-occurrence images depicting predator–pest pairs in natural environments, often with occlusions and complex backgrounds, suitable for ecological relation modeling; and (3) pest-dense images for training detection and localization modules. All images were resized to 224 × 224 and manually annotated by three entomology experts using LabelMe and CVAT. Bounding boxes and class labels were provided for each insect instance, resulting in a high-quality, structured dataset comprising 5037 images across eight major categories. This construction ensures both semantic diversity and balanced category distribution for robust model training.

### 3.2. Data Preprocessing and Augmentation

During the training of object detection models, raw image data often exhibit issues such as varying dimensions, uneven feature distributions, and imbalanced object occurrences. These factors may significantly affect the convergence rate and ultimate performance of the model. To enhance the robustness and generalization ability of the detection system, preprocessing and augmentation of image data have become indispensable components. By standardizing image dimensions, incorporating diverse augmentation strategies, and designing specific enhancements for small objects, the diversity of training samples can be increased, while simultaneously mitigating the model’s dependence on specific image structures. This ultimately improves the model’s performance in real-world applications. To ensure consistent input to the model, all raw images are first resized to a fixed resolution (e.g., 512×512 or 640×640) prior to being fed into the neural network. This uniform resizing not only accelerates batch processing during training but also provides structural advantages for subsequent convolution operations. Let the original image size be H×W and the target resolution be $H^{\prime }\times W^{\prime }$; then, the resizing operation can be represented as follows:

(1) $$I^{\prime }=\mathrm{Resize}\left(I,H^{\prime },W^{\prime }\right),$$

where *I* denotes the original image, $I^{\prime }$ represents the resized image, and Resize(·) denotes the resizing operation that preserves the aspect ratio or applies padding if necessary. This step establishes a standardized foundation for subsequent augmentation procedures. To further improve the model’s robustness in complex environments, two mainstream data augmentation methods, CutMix and GridMask, are incorporated in this study. CutMix involves randomly mixing two images and adjusting their corresponding bounding boxes and labels accordingly. This strategy enhances the model’s adaptability to occlusion and partial object loss. The operation proceeds as follows: a rectangular region is randomly cropped from image *A* and pasted onto image *B*, with the associated labels weighted according to the area ratio. The formulation is given by

(2) $$\tilde{x}=M⊙x_{A}+\left(1-M\right)⊙x_{B},$$

In this equation, $x_{A}$ and $x_{B}$ are the original images *A* and *B*, respectively, *M* is the binary mask matrix, and ⊙ denotes the Hadamard product (element-wise multiplication). This method improves the model’s ability to learn from various spatial composition patterns. GridMask, on the other hand, is a structured occlusion method that simulates occlusion noise by masking specific regions in a grid pattern, thereby enhancing the model’s discriminative capability. A periodic grid mask is applied to the image, defined as follows:

(3) $$\tilde{x}=x⊙\left(1-G\right),$$

where *x* denotes the original image and *G* is the periodic mask matrix. Unlike random occlusion, GridMask enforces stronger spatial distribution constraints, allowing the model to maintain a global judgment ability despite localized feature loss. Given the difficulty of detecting small objects in object detection tasks, a small-object enhancement module is further introduced, along with the construction of a multi-scale image pyramid, to improve the model’s perception of small-scale targets. Small objects, due to their sparse pixel information and strong background interference, are often challenging for deep feature extraction modules to capture effectively. To address this, small-object duplication and scaling enhancements are applied during preprocessing. Regions containing small objects are extracted from the original image, duplicated at different positions, or scaled to larger sizes before being pasted back. This significantly enhances the prominence of small objects in the training data. The procedure is expressed as follows:

(4) $$I^{\prime }=I+\sum_{i=1}^{n}\mathrm{Scale}\left(\mathrm{Crop}\left(I,b_{i}\right),s\right),$$

where $\mathrm{Crop}(I,b_{i})$ denotes the extraction of a target region from image *I* based on bounding box $b_{i}$, Scale(·,s) represents the scaling operation with scaling factor *s*, and *n* is the number of small objects being augmented. Building upon this, an image pyramid is constructed to simulate multi-scale inputs, thereby enhancing the network’s ability to represent objects at various scales. The multi-scale image pyramid downsamples the original image to multiple resolutions and inputs them into different perceptual layers of the model. By retaining fine-grained information of small objects in shallow layers, the model can more comprehensively capture spatial features of targets at different scales. This process is formulated as

(5) $$P=\mathrm{Resize}\left(I,s_{k}\right)|s_{k}\in S,$$

where P denotes the set of image pyramids, *S* is the set of scaling factors (e.g., 0.5,1.0,1.5), and each $s_{k}$ corresponds to a specific scaling ratio. In this study, preprocessing and augmentation of image data play a critical role. Standardizing image resolution ensures input normalization, while CutMix and GridMask significantly enhance the model’s robustness under complex conditions. Moreover, the small-object enhancement module and multi-scale image pyramid substantially improve the model’s detection capability for small-sized targets. These strategies collectively establish a solid data foundation for constructing an object detection system with a strong generalization ability and high detection accuracy.

### 3.3. Proposed Method

#### 3.3.1. Overall

**Figure 2:**
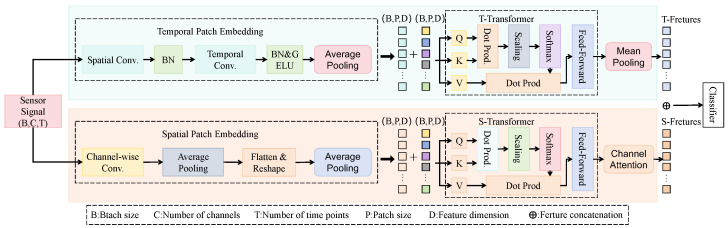
Overall architecture of the proposed model. The input sensor signals are first processed through spatial and temporal convolutional layers to extract local features, followed by spatial and temporal patch embedding to obtain unified feature dimensions. These are then fed into the temporal Transformer (T-Transformer) and spatial Transformer (S-Transformer) to capture global dependencies across time and channels, respectively. Here, B, C, and T denote batch size, number of channels, and number of time points, respectively; P indicates patch size and D denotes feature dimension.

The proposed model adopts a modular hybrid architecture, forming a closed-loop pipeline of multi-object recognition, co-occurrence modeling, and relationship inference. The entire process begins with an input image, which sequentially passes through four stages, local feature extraction, global relationship modeling, label attention interaction, and pairwise label prediction, as shown in Figure 2. Specifically, the input image is first processed by the convolutional module, where multiple spatial convolutional layers are utilized to extract local texture features. These features are then enhanced through batch normalization and nonlinear activation functions. The intermediate outputs from this stage preserve rich hierarchical spatial information and are capable of capturing critical details such as insect contours and color patterns.

Next, the feature maps are divided into spatial patches and reshaped into sequential embeddings, which are then fed into the spatial Transformer module (S-Transformer). This module employs multi-head attention mechanisms to model long-range contextual dependencies across different image regions. During this process, the spatial interaction semantics among insect targets are encoded into high-dimensional global representations, which facilitate accurate boundary identification in scenarios involving occlusion or dense object distribution. Subsequently, the global image features are combined with predefined label embeddings and input into the multi-label co-attention module. This module constructs an interaction matrix between image semantics and label space, enabling the learning of co-occurrence relations among insect categories and guiding the image feature refinement for pairwise prediction enhancement. In particular, each label embedding is matched with image features based on similarity, and the co-occurrence probability distribution among labels is calculated using the attention mechanism. This design allows the model to provide stronger interpretability when recognizing combinations such as “chrysopid-thrips” or “ladybug-aphid”. The resulting multi-label embeddings are further processed through channel-wise attention fusion, feature concatenation, and pooling operations, before being passed to the final classifier, which outputs the prediction probability for each insect category. Additionally, a pairwise label matching loss function is introduced during training to explicitly supervise the model’s ability to distinguish valid “predator–host” label pairs. In contrast to conventional approaches that treat each label independently, this mechanism enables the explicit modeling of semantic dependencies among categories, thereby enhancing the model’s capability to understand compound biological relationships. The overall structure successfully bridges the gap between single-object recognition and multi-object relational modeling, maintaining inference efficiency while offering strong ecological interpretability and practical deployment potential.

#### 3.3.2. Multi-Label Co-Attention Module

The multi-label co-attention module proposed in this study is designed to enhance the semantic modeling of pairwise relationships between predators and pests. Its core principle differs from conventional self-attention mechanisms, which primarily model internal dependencies within a single set of input features by computing self-matching across all spatial positions to capture contextual structures. In contrast, the co-attention mechanism establishes a cross-attention pathway between image features and label embeddings, focusing on modeling co-occurrence relationships among labels and guiding semantic reasoning over the image. Fundamentally, it constitutes a heterogeneous interactive attention mechanism that learns the matching weights between image content and label semantics, thereby improving the recognition performance of target pairs. This design is particularly well-suited to biological control systems in agricultural scenarios, where explicit ecological pairing structures, such as predator–host relationships, are common and essential.

**Figure 3:**
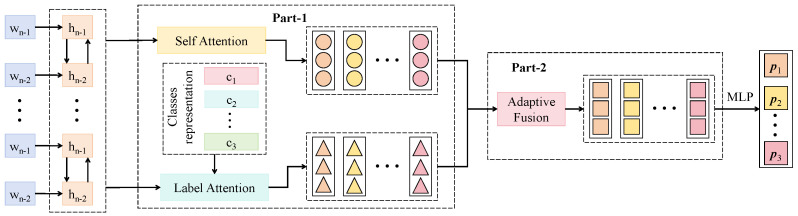
This figure illustrates the architecture of the multi-label co-attention module. The module consists of two main parts: Part-1 performs bidirectional attention interaction between image features and label embeddings, enabling adaptive fusion of visual and semantic information; Part-2 employs a multi-layer perceptron (MLP) for multi-label prediction based on the fused representations. The incorporation of dual-path self attention and label attention mechanisms significantly enhances the model’s ability to capture co-occurrence relationships between predators and pests.

The architecture of this module, as shown in Figure 3, consists of two main parts: image–label semantic interaction encoding (Part-1) and attention-based prediction fusion (Part-2). The input image features, extracted from the preceding backbone module, have a shape of [B,C,H,W], where B=32 (batch size), C=256 (channels), and H=W=14. The label embedding matrix is initialized with shape $\left[C_{L},d\right]$, where $C_{L}=8$ represents the number of label categories and d=256 is the embedding dimension. The image features are then downsampled via average pooling and flattening into a sequence of size [B,N,d], with N=H×W=196. The label embeddings are extended with positional encoding and expanded to $\left[B,C_{L},d\right]$, and both embeddings are passed into the cross-attention module, where the attention score is computed as follows:

(6) $${\mathrm{Att}}_{ij}=\frac{\left(e_{i}W_{Q}\right){\left(f_{j}W_{K}\right)}^{⊤}}{\sqrt{d}},$$

where $e_{i}\in R^{1\times d}$ is the *i*-th label embedding, $f_{j}\in R^{1\times d}$ is the *j*-th image patch feature, and $W_{Q},W_{K}\in R^{d\times d}$ are the query and key projection matrices, respectively. The resulting attention scores between each label and image patch are then used as weights to re-aggregate the image features and generate label-aware semantic vectors:

(7) $$z_{i}=\sum_{j=1}^{N}\mathrm{softmax}\left({\mathrm{Att}}_{ij}\right)\cdot \left(f_{j}W_{V}\right),$$

where $W_{V}\in R^{d\times d}$ is the value transformation matrix. This operation functions as label-guided semantic filtering over the image, extracting spatial regions that are highly correlated with each label. The resulting vectors $Z_{i}$ are then passed through a two-layer MLP with hidden sizes of 512 and 256, respectively, and fused with the original label embeddings $E_{i}^{l}$ via residual connections to produce the final label-aware representations ${\hat{E}}_{i}$. The final output has a shape $\left[B,C_{L}\right]$, representing the presence probabilities for each insect category. This module is jointly applied with the global Transformer structure in the backbone network. While the Transformer models contextual relationships between image regions, the co-attention module focuses on reasoning over interactions among labels. The motivation for this dual-stream design lies in the limitation of spatial dependency modeling alone, which cannot explicitly capture the ecological co-occurrence structures among insect targets. By contrast, the label embedding space, equipped with attention mechanisms, facilitates the construction of stable semantic graphs, thereby improving the model’s capacity to make joint predictions of target pairs. During training, the parameters of the co-attention module are updated jointly with the backbone using the composite loss function $L_{total}$, ensuring a closed-loop enhancement between image–label relationships and image region semantics. Empirical results demonstrate that this module significantly improves the model’s ability to recognize common biological combinations such as ladybug–aphid and orius–thrips, especially under complex background conditions or when targets are visually indistinct, thus contributing to the robustness and ecological interpretability of the overall system.

#### 3.3.3. Pairwise Label Matching Loss

**Figure 4:**
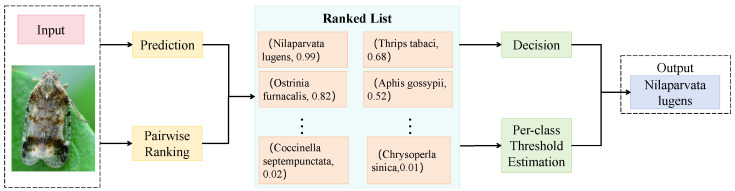
Illustration of the pairwise label matching loss. The figure depicts the modeling process for capturing pairwise label relations in a multi-label recognition task. Initially, the backbone network outputs confidence scores for each insect category, followed by the construction of a ranked list based on these scores. Pairwise similarity is then computed across label pairs and integrated with a per-class threshold estimation module to determine matched pairs dynamically. This mechanism enhances the model’s capability to learn semantic co-occurrence relations among labels, thereby improving the precision of ecological pairing recognition.

In conventional multi-label classification tasks, binary cross-entropy (BCE) loss is typically employed to independently model each label, treating the presence or absence of every label as an isolated binary classification task. However, in the context of predator–pest recognition in agriculture, strong symbiotic or mutually exclusive relationships frequently exist between target labels, particularly between specific natural enemies and their corresponding pest species. The traditional BCE loss fails to explicitly model these inter-label dependencies, which may result in semantic conflicts or the omission of pairwise structures when addressing co-occurrence prediction tasks, thereby limiting the ecological interpretability of the system. To overcome this limitation, a pairwise label matching loss is proposed in this study to explicitly model the collaborative dependencies between label pairs during optimization and to improve the model’s capability to discriminate between ecologically valid insect combinations. As shown in Figure 4, the central idea of this loss function is to construct pairwise prediction probabilities between labels to represent whether two categories appear in the image as a meaningful combination. Given an input image x and its corresponding ground-truth label set Y⊆1,2,…,C, where *C* denotes the total number of classes, the co-occurrence label indicator for each pair (i,j) is defined as follows:

(8) $$y_{ij}=\left\{\begin{array}{cc} 1, & \mathrm{if}\;i\in Y,j\in Y\\ 0, & \mathrm{otherwise} \end{array}\right.$$

Given the predicted multi-label probabilities ${\hat{p}}_{i}\in \left[0,1\right]$, the joint prediction probability of a label pair is approximated as ${\hat{p}}_{ij}={\hat{p}}_{i}\cdot {\hat{p}}_{j}$, and the proposed pairwise label matching loss is formulated as follows:

(9) $$L_{\mathrm{pair}}=\sum_{(i,j)\in P}\left(y_{ij}\cdot \log\left({\hat{p}}_{i}\cdot {\hat{p}}_{j}\right)+\left(1-y_{ij}\right)\cdot \log\left(1-{\hat{p}}_{i}\cdot {\hat{p}}_{j}\right)\right),$$

where P denotes the set of all candidate label pairs. This formulation can be interpreted as BCE loss over the joint probabilities of label pairs, explicitly penalizing incorrect predictions on pairwise combinations. Unlike the traditional BCE loss that focuses solely on optimizing individual label predictions ${\hat{p}}_{i}$, the pairwise loss redirects the optimization objective toward joint modeling of label interactions, thereby enhancing the accuracy of predicting ecologically valid pairs such as “ladybug–aphid” and “chrysopid–thrips.” This mechanism encourages the model to learn the latent co-occurrence patterns among labels and to encode inter-label semantic structures into the final output representations. Moreover, the pairwise loss exhibits a theoretical capacity to model label dependencies. When each predicted label score is regarded as an independent Bernoulli-distributed variable $p_{i}$, the expected value of the joint co-occurrence probability can be expressed as $E\left[{\hat{p}}_{i}\cdot {\hat{p}}_{j}\right]=E\left[{\hat{p}}_{i}\right]E\left[{\hat{p}}_{j}\right]+\mathrm{Cov}\left({\hat{p}}_{i},{\hat{p}}_{j}\right)$. During optimization with the pairwise loss, the model is encouraged to learn positive covariance $\mathrm{Cov}({\hat{p}}_{i},{\hat{p}}_{j})>0$ for true co-occurring pairs, reflecting statistically positive relationships and fostering stable attention pathways for label pairing. Conversely, for label pairs that do not co-occur, the model learns to suppress spurious combinations by minimizing ${\hat{p}}_{i}\cdot {\hat{p}}_{j}$. In the context of this study, the proposed loss is jointly optimized with the standard multi-label BCE loss as follows:

(10) $$L_{\mathrm{total}}=L_{\mathrm{bce}}+\lambda \cdot L_{\mathrm{pair}},$$

where λ denotes a weighting coefficient. In the experiments, λ is set to 0.4, which effectively balances classification accuracy and the precision of ecological pairing. This method is particularly suitable for agricultural pest control scenarios characterized by highly sparse label pair distributions and strong semantic dependencies. It not only improves the model’s robustness in complex multi-object settings but also enhances the interpretability of ecological decision making. When combined with the proposed label co-attention mechanism, the model establishes a tri-level inference structure encompassing image, label, and ecological pair representations, enabling joint reasoning over multiple targets and supporting scalable applications in agricultural intelligent recognition systems.

### 3.4. Experimental Design

#### 3.4.1. Evaluation Metrics

To comprehensively evaluate the performance of the proposed model in multi-label classification and pairwise matching tasks, four commonly used quantitative metrics were adopted: precision, recall, F1-score, and mean average precision (mAP). The formulations are defined as follows:

(11) $$\begin{array}{cc} \mathrm{Precision} & =\frac{TP}{TP+FP},\\ \mathrm{Recall} & =\frac{TP}{TP+FN},\\ F1\text{-}\mathrm{Score} & =\frac{2\cdot \mathrm{Precision}\cdot \mathrm{Recall}}{\mathrm{Precision}+\mathrm{Recall}},\\ \mathrm{mAP}@k & =\frac{1}{N}\sum_{i=1}^{N}{\mathrm{AP}}_{i}@k \end{array}$$

In the above equations, TP (true positive) denotes the number of correctly predicted positive labels, FP (false positive) represents the number of incorrectly predicted positive labels, and FN (false negative) indicates the number of relevant labels that were not predicted. Precision reflects the proportion of correctly predicted positive labels among all predicted positive labels, whereas recall measures the proportion of correctly predicted positive labels among all ground-truth positive labels. F1-score serves as the harmonic mean of precision and recall, balancing both metrics. The mean average precision at threshold *k* (denoted as mAP@*k*) is computed by averaging the individual average precision values ${\mathrm{AP}}_{i}$ across all *N* categories at a given Intersection over Union (IoU) threshold *k*, commonly set at 50% and 75%. These metrics together offer a holistic assessment of model performance, capturing both exactness (precision) and completeness (recall), while F1-score and mAP provide summary-level indicators for comparative evaluation across models and configurations.

#### 3.4.2. Baseline Methods

To comprehensively evaluate the effectiveness of the proposed method, seven representative models were selected as baseline comparisons. These include classical convolutional architectures, state-of-the-art detection frameworks, and Transformer-based models, spanning diverse structural paradigms in multi-label image recognition.

The ResNet-50 [35] multi-label classification model, as a classical CNN representative, exhibits stable training performance and robust local feature extraction capabilities. In this study, the softmax classifier was replaced with a sigmoid-activated multi-label output layer, and binary cross-entropy was used as the loss function, enabling independent label prediction suitable for agricultural imagery. The YOLOv8 [36] model integrated with a multi-label head retains the high-speed detection of the original YOLO framework while introducing a parallel semantic prediction branch. This joint modeling of object localization and label classification enhances performance in field images where multiple pest instances co-exist. YOLOv10 [37] further strengthens the backbone and label modeling through deeper convolutional layers and a dynamic multi-label regression strategy, leading to improved expressiveness in high-complexity environments. YOLOv11 [38], as an upgraded version of the YOLO series, incorporates an enhanced decoder and semantic-aware modules. It demonstrates superior label discrimination capabilities and precision in handling dense pest clusters and complex background interference. RetinaDet [39] combines an anchor-based mechanism with a feature pyramid network (FPN), offering excellent multi-scale perception. This architecture enables stable multi-label prediction, especially for objects with large scale variations in agricultural imagery. DETR [40] employs a Transformer encoder for image sequence modeling and performs object detection in an end-to-end manner. Its self-attention mechanism enables effective modeling of semantic dependencies among labels, performing well in multi-object co-occurrence scenarios. The Vision Transformer (ViT) [41] model divides images into patch sequences and processes them through stacked Transformer encoders to capture global semantic context. Its strong capacity for long-range dependency modeling makes it particularly advantageous in scenarios with high label co-occurrence and semantic overlap, such as multi-label pest recognition.

#### 3.4.3. Platform and Environment

To systematically validate the effectiveness, generalization, and adaptability of the proposed multi-label image recognition method under complex label structures, experiments were conducted on a high-performance computing platform equipped with abundant software and hardware resources. The experimental hardware included a deep learning server configured with an NVIDIA A100 GPU (80 GB memory), Manufacturer: NVIDIA Corporation, Santa Clara, CA, USA, an Intel Xeon Gold 6248R processor (Manufacturer: Intel Corporation, Santa Clara, CA, USA), and 512 GB of RAM, enabling large-scale image processing, deep neural network training, and multi-task parallel execution. The operating system was Ubuntu 20.04 LTS, providing a stable Linux environment for efficient deployment of deep learning toolchains. The primary software framework used was PyTorch 2.0, integrated with CUDA 11.8 and cuDNN 8.6 to fully exploit GPU parallelism, thereby accelerating both forward and backward propagation processes. Python 3.9 served as the main programming language, supported by commonly used scientific computing and visualization packages such as NumPy, Pandas, OpenCV, Matplotlib, and Seaborn, which facilitated data loading, preprocessing, model monitoring, and performance visualization. The Scikit-learn library was utilized for computing evaluation metrics, constructing confusion matrices, and performing K-fold cross-validation, enhancing the statistical rigor of the experiments. For training configuration, the AdamW optimizer was employed, introducing weight decay into the traditional Adam framework to better control overfitting while maintaining convergence efficiency. The initial learning rate was set to $1\times 10^{-4}$ and adjusted using a cosine annealing strategy, which gradually decayed the learning rate to improve training stability and generalization in later stages. Each training epoch was configured with a batch size of 32, and gradient accumulation was adopted to effectively increase the number of samples per optimization step while maintaining reasonable memory usage. To further accelerate training and reduce memory consumption, mixed precision training was implemented, combining FP16 and FP32 computations to enhance overall computational efficiency. An early stopping strategy was introduced, terminating training if no significant improvement in validation loss was observed over 10 consecutive epochs. This approach prevented overfitting in later training stages and conserved computational resources. The dataset was split into training and validation sets at a 7:3 ratio based on the original label distribution, ensuring adequate sample support for training while preserving evaluation objectivity. To improve evaluation robustness, the main experiments were repeated five times, and the average of key metrics was reported to mitigate performance fluctuations caused by dataset partitioning.

## 4. Results

### 4.1. Performance Comparison with Baseline Methods on the Test Set

This experiment aims to evaluate the effectiveness of the proposed hybrid CNN-Transformer model against seven representative baselines, including ResNet-50, the YOLO series (v8–v11), RetinaDet, DETR, and Vision Transformer (ViT). These models span a diverse set of architectural paradigms, ranging from conventional convolutional classifiers to detection-based and Transformer-based frameworks. Performance was assessed using standard multi-label metrics—precision, recall, and F1-score—along with mAP@50 and mAP@75 to reflect both classification and localization capabilities under complex field conditions. Notably, our model achieved an F1-score of 86.5%, with mAP@50 and mAP@75 scores of 85.1% and 73.8%, respectively. Compared to the strongest baseline (ViT, 80.9% F1), our method yields a relative improvement of +5.6% in F1, +5.7% in mAP@50, and +5.7% in mAP@75. More impressively, the model achieved a generalization F1-score of 79.6% on previously unseen predator–pest combinations, underscoring its ecological robustness and transferability beyond the training distribution.

**Figure 5:**
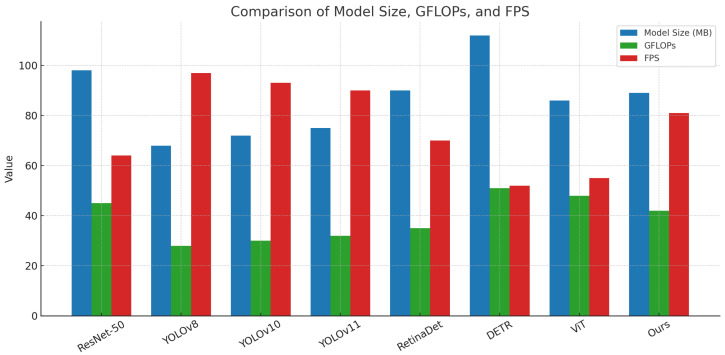
Efficiency comparison with baseline methods on the test set.

**Figure 6:**
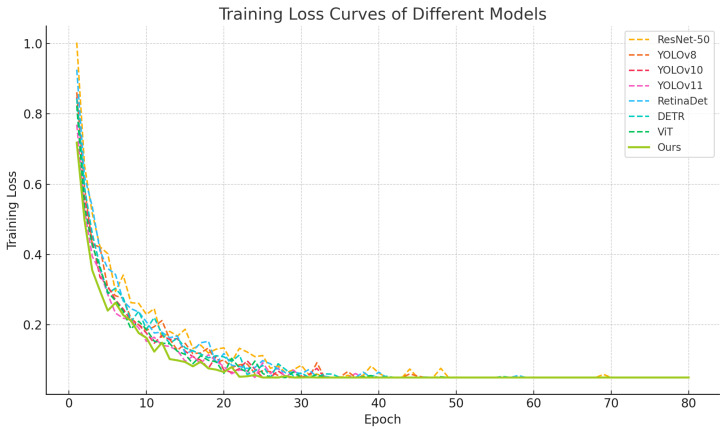
Training curve with baseline methods on the test set.

**Figure 7:**
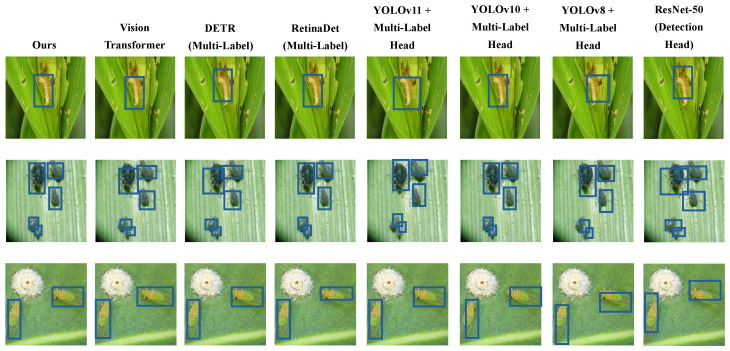
Detection visualization of different methods.

As shown in Table 2 and Figure 5, Figure 6 and Figure 7, relatively lower performance was observed for ResNet-50 across all metrics due to its shallow convolutional structure and independent label prediction strategy. A progressive improvement in detection and label recognition was observed from YOLOv8 to YOLOv11, with YOLOv11 achieving an F1-score of 81.9, attributed to its deeper decoding layers which better handle dense object scenarios. RetinaDet demonstrated strong detection accuracy but slightly underperformed in recall and F1-score, possibly due to limitations of the anchor mechanism in detecting small objects. DETR, as a Transformer-based end-to-end detector, exhibited robust label coupling modeling capabilities through its self-attention mechanism. ViT achieved high classification performance in complex images due to its global contextual modeling. The proposed model, which integrates local texture extraction of CNNs with global dependency modeling of Transformers, outperformed all baselines across all metrics, with a notable lead in mAP@75, reflecting superior fine-grained object recognition and pairing prediction. From a mathematical perspective, the inclusion of a co-occurrence attention mechanism and a pairwise matching loss function enabled effective learning of joint semantic distributions among labels in high-dimensional feature space, thereby enhancing recognition accuracy and ecological interpretability.

### 4.2. Ablation Study on Attention Mechanisms

This experiment was designed to investigate the impact of different attention mechanisms on multi-label recognition performance, with the objective of validating the effectiveness of the proposed co-occurrence attention mechanism in modeling predator–pest pairing relationships. Three experimental configurations were evaluated: a baseline model without attention, a model employing conventional self-attention, and a model integrating the proposed co-attention mechanism. By comparing these variants across multiple evaluation metrics, the role of the attention module in the overall recognition framework was assessed. This study provides both theoretical insights and empirical evidence regarding the contribution of inter-label interaction modeling to multi-label prediction tasks.

**Figure 8:**
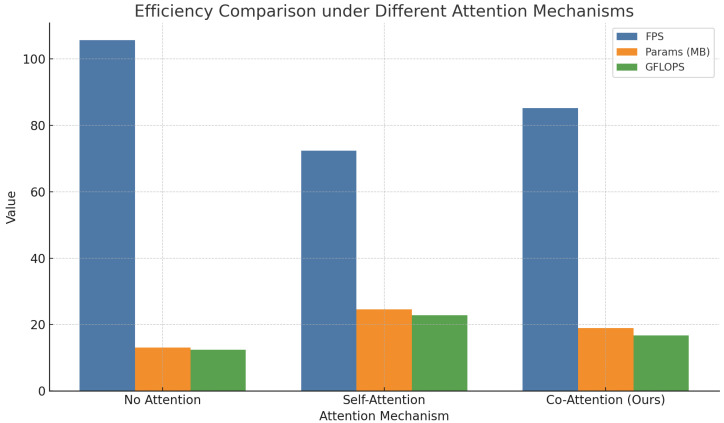
Visualization of different attention mechanisms.

As shown in Table 3 and Figure 8, the model without any attention mechanism demonstrates a certain level of baseline recognition ability, but its performance in terms of recall and mAP is limited due to the lack of context-aware label interactions. When self-attention is incorporated, the model is able to dynamically reweigh spatial features, resulting in improved overall performance, particularly in precision. However, because self-attention is restricted to dependencies within the image domain, it fails to fully capture semantic relationships between labels, limiting its effectiveness. In contrast, the proposed co-attention mechanism addresses both image-level and label-level semantics by introducing structured similarity modeling between label embeddings. Mathematically, the co-attention module constructs bidirectional interaction paths between image features and label embeddings, generating dynamic semantic representation matrices. This design enables effective learning and propagation of attention distributions across labels, significantly enhancing the model’s robustness in recognizing paired targets. The integration of this module yields notable improvements in F1-score and mAP, thereby confirming its superior adaptability and theoretical validity in agricultural ecological recognition tasks.

### 4.3. Generalization Evaluation on Unseen Predator–Pest Combinations

This experiment was designed to evaluate the model’s generalization capability when encountering predator–pest combinations that were not included during training. The objective was to determine whether the model possesses the capacity to transfer learned pairing structures to new ecological contexts. Several predator–pest pairs, which were ecologically plausible but excluded from the training set, were constructed and used in the test phase to observe the model’s performance in identifying and matching these previously unseen combinations. Precision, recall, and F1-score were used as quantitative metrics for evaluation.

As shown in Table 4, all unseen combinations maintained an F1-score above 76%, with the pair *C. sinica* and *N. lugens* achieving the highest score of 82.3%, indicating the model’s strong adaptability to novel ecological pairings. These results suggest that the model does not merely rely on memorization of training samples but instead learns transferable semantic representations of label pairing through the integration of attention-based modeling and pairwise loss. Mathematically, the co-attention module constructs a latent pairing space by capturing similarity among label embeddings, allowing semantic vector representations to become composable and additive. When presented with unseen combinations, the model leverages the learned feature distributions of predators and pests to infer plausible ecological associations and produce corresponding predictions. Additionally, the pairwise label matching loss strengthens the model’s capacity to model joint distributions of label pairs, shifting the prediction paradigm from independent classification toward integrated ecological inference. These findings confirm that the proposed method possesses a robust inter-class generalization ability, offering a practical value for future biological control recommendations.

## 5. Discussion

### 5.1. Practical Applications and Ecological Value of the Proposed Model

The proposed hybrid CNN-Transformer model achieves high accuracy and demonstrates generalization capability in multi-label recognition tasks focused on predator–pest co-occurrence in agricultural imagery. Notably, the model maintains stable performance even in complex visual environments or when recognizing previously unseen insect combinations, achieving an average F1-score of 79.6% on such pairs. This level of robustness can help reduce manual inspection workloads, which typically require 2–3 h of daily monitoring per hectare during peak pest periods. For example, in vegetable cultivation zones where Orius sauteri is commonly used to suppress pests such as thrips and aphids, the model enables automatic identification and quantification of predator and pest presence in co-located imagery. Such automation—deployable via fixed camera stations or UAV platforms—can reduce dependence on labor-intensive field surveys and support more timely and consistent biocontrol assessments. In rice production regions where outbreaks of *N. lugens* frequently lead to pesticide overuse, the ability to detect natural enemies like *T. chilonis* and *C. sinica* and to map their co-occurrence with pests may aid in decision making for targeted ecological interventions. Similarly, in ecological corridors and intercropped fields where insect distributions are spatially variable, the model’s multi-target recognition capacity can support the generation of localized pest pressure maps, aiding fine-scale pest management planning. It is important to acknowledge the computational trade-offs involved. The current model processes an image in approximately 120 milliseconds on an NVIDIA A100 GPU, which is suitable for server-based analysis but may exceed the capabilities of edge devices without optimization. To address this, future work will explore model compression techniques such as pruning and quantization, with the aim of enabling real-time inference on embedded systems. Given ongoing progress in both algorithmic efficiency and agricultural sensing infrastructure, we anticipate that pilot deployments in commercial field environments could be feasible within one to two growing seasons in regions equipped with UAV or smart monitoring platforms.

### 5.2. Limitation and Future Work

While the proposed hybrid CNN-Transformer multi-label recognition model has demonstrated strong performance in detecting predator–pest co-occurrences in agricultural imagery, several limitations remain that warrant further attention. First, the current model has been trained and evaluated primarily on static images captured under relatively favorable weather and lighting conditions. In real-world agricultural settings, factors such as rain, fog, dust, or poor illumination could significantly degrade image quality and impair the model’s ability to correctly identify small or partially occluded insects. Addressing this issue will require future work in developing robustness-enhancing techniques, such as data augmentation with synthetic weather effects or the integration of infrared imaging. Second, the model’s inference speed—approximately 120 milliseconds per image on an NVIDIA A100 GPU—is not yet suitable for direct deployment on low-power edge devices common in agricultural fields. Real-time applications, particularly those involving UAVs or in situ camera systems, will demand further optimization through model compression techniques, such as pruning, quantization, or knowledge distillation, in order to reduce computational and memory requirements while maintaining recognition accuracy. Third, the dataset used for model training was collected from selected agricultural zones in China. While it provides ecologically valid data for the local context, insect species, crop types, and predator–prey relationships can vary significantly across regions and climates. As a result, the current model may not generalize well to global agricultural systems without domain adaptation. Future work should include cross-regional data collection and transfer learning approaches to extend the model’s applicability to diverse agroecological settings. Looking forward, several concrete research directions are being considered. One is the incorporation of temporal dynamics through video-based input to analyze behavioral patterns and predator–prey interactions over time, thereby enhancing ecological inference. Another is the adaptation of the model to region-specific pest management practices by fine-tuning on locally annotated datasets. Moreover, integrating the visual detection framework with environmental sensor networks—such as temperature, humidity, or pheromone trap data—could enable the development of a multimodal intelligent pest monitoring system, supporting proactive and precise biocontrol interventions. These steps will contribute toward realizing practical, scalable, and globally applicable solutions for sustainable pest management in agriculture.

## 6. Conclusions

This study addresses the growing need for intelligent biological control in the context of sustainable agriculture by proposing a multi-label recognition model that integrates CNNs with Transformer-based architectures. The model is designed to identify and predict co-occurrences of natural enemies and pests in field imagery. Structurally, it combines the local texture extraction capabilities of CNNs with the global dependency modeling strengths of Transformers. A multi-label co-occurrence attention mechanism is introduced to enhance semantic interactions among labels, while a pairwise label matching loss is formulated to improve the accuracy of ecological pair predictions. Across multiple experiments, the proposed method demonstrates significant performance advantages over baseline models such as ResNet, YOLOv8, and ViT in metrics including mAP50, F1-score, precision, and recall. Specifically, an F1-score of 86.5 and an mAP75 of 73.8 were achieved, highlighting the model’s strong adaptability to complex multi-object scenarios. Notably, the model maintained an average F1-score of 79.6 in tests involving previously unseen predator–pest combinations, confirming its transferability in practical ecological applications. Additionally, the first annotated image-pairing dataset for predator–pest combinations was constructed, and an end-to-end recognition framework was established—ranging from visual detection to ecological modeling. These contributions provide both technical support and theoretical foundations for advancing biological pest control toward intelligent, data-driven agricultural systems.

## Figures and Tables

**Table 1:** Annotated image statistics for natural enemies and pests.

Category	Type	Image Count	Co-Occurrence Samples
*C. septempunctata*	Predator	642	412
*C. sinica*	Predator	711	385
*O. sauteri*	Predator	538	304
*T. chilonis*	Predator	483	271
*A. gossypii*	Pest	923	513
*T. tabaci*	Pest	786	448
*O. furnacalis*	Pest	512	276
*N. lugens*	Pest	442	199
**Total**	-	**5037**	**2808**

**Table 2:** Performance comparison with baseline methods on the test set (relative gains over best baseline in parentheses).

Model	Precision (%)	Recall (%)	F1-Score (%)	mAP@50 (%)	mAP@75 (%)
ResNet-50 (Detection Head)	78.2	74.9	76.5	73.1	62.4
YOLOv8 + Multi-Label Head	81.5	76.3	78.8	76.9	65.7
YOLOv10 + Multi-Label Head	83.1	77.9	80.4	78.6	66.9
YOLOv11 + Multi-Label Head	84.5	79.5	81.9	80.2	68.4
RetinaDet (Multi-Label)	79.8	75.1	77.4	74.5	63.7
DETR (Multi-Label)	82.4	77.3	79.8	77.9	67.2
Vision Transformer (ViT)	83.6	78.4	80.9	79.4	68.1
**Ours**	**88.9 (+5.3%)**	**84.2 (+5.8%)**	**86.5 (+5.6%)**	**85.1 (+5.7%)**	**73.8 (+5.7%)**

**Table 3:** Ablation study results on attention mechanisms.

Attention Mechanism	Precision (%)	Recall (%)	F1-Score (%)	mAP@50 (%)	mAP@75 (%)
No Attention	82.4	77.6	79.9	76.3	65.0
Self-Attention	85.7	80.5	83.0	80.1	68.7
Co-Attention (Ours)	**88.9**	**84.2**	**86.5**	**85.1**	**73.8**

**Table 4:** Generalization evaluation on unseen predator–pest combinations.

Unseen Pair Type	Precision (%)	Recall (%)	F1-Score (%)
*T. chilonis*-*T. tabaci*	81.2	76.0	78.5
*O. sauteri*-*N. lugens*	83.6	78.4	80.9
*C. septempunctata*-*O. furnacalis*	79.8	74.2	76.9
*C. sinica*-*N. lugens*	84.4	80.3	82.3
Average	**82.3**	**77.2**	**79.6**

## Data Availability

The data presented in this study are available upon request from the corresponding author.
